# Buttress plating versus trans-syndesmotic stabilisation for posterolateral malleolus fractures: outcomes with early weight-bearing

**DOI:** 10.1007/s00590-026-04904-w

**Published:** 2026-08-28

**Authors:** Katie Hutchinson, Georgina Crate, Christopher Fenner, Alex Trompeter, Dominic Nielsen

**Affiliations:** 1 St George’s University Hospitals NHS Foundation Trust, London, UK; 2 Kingston Hospital NHS Trust, London, UK; 3 St George’s, University of London, London, UK

**Keywords:** Posterior malleolus, Syndesmosis, Buttress, Weightbearing

## Abstract

**Purpose:**

Posterolateral posterior malleolar fractures are commonly associated with syndesmotic instability, yet the optimal fixation strategy remains unclear. This study compared postoperative complications following direct posterior buttress plating and indirect trans-syndesmotic stabilisation, and evaluated differences in postoperative weight-bearing.

**Methods:**

A retrospective review of a prospectively maintained trauma database at a Level 1 major trauma centre (2012–2021) was performed. Patients with posterior malleolar fractures treated with buttress plating or syndesmotic stabilisation were included. Radiographs, with computed tomography where available, were used to assess fragment size. Postoperative complications and factors associated with early weight-bearing were analysed using multivariable logistic regression.

**Results:**

Of 1283 ankle fractures, 445 (34.6%) involved the posterior malleolus; 382 met the inclusion criteria. Buttress plating was performed in 184 patients and syndesmotic stabilisation in 111. Mean follow-up was 10 and 8.8 months. Overall complication rates were similar, with no significant differences in infection, loss of reduction, revision surgery, or arthrodesis. Metalwork removal was more frequent following syndesmotic stabilisation (37.8% vs 10.3%, *p* < 0.001). Early weight-bearing was more common after buttress plating (41.8% vs 22.9%) and remained independently associated after adjustment (OR 2.81, 95% CI 1.12–11.46).

**Conclusion:**

Both techniques demonstrate comparable safety profiles. However, direct fixation was associated with a greater likelihood of early postoperative weight-bearing. As postoperative rehabilitation protocols were not standardised, this finding should be interpreted cautiously and may reflect surgeon preference in addition to fixation strategy. In the presence of a syndesmotic injury associated with a posterior malleolar fragment of sufficient size to permit fixation, direct fixation of the posterior malleolar fragment should be considered, as it may restore stability and facilitate rehabilitation. Further prospective studies with standardised rehabilitation protocols and longer-term follow-up are required.

## Introduction

Ankle fractures are common injuries, with an annual incidence of approximately 75 per 100,000 population, and involve the posterior malleolus in up to 44% of cases [1]. Posterior malleolar fractures are a heterogeneous group, encompassing several fracture patterns with differing implications for ankle stability. Traditionally, the decision to fix the posterior malleolus was guided by fragment size, with involvement of ≥ 20% of the articular surface commonly used as a threshold for surgery [2]. However, advanced imaging has shown that fracture configuration, rather than fragment size alone, is a stronger determinant of stability and outcome [3].

Posterolateral (Volkmann) fragments are particularly important, as they are frequently associated with syndesmotic disruption and require careful reduction to restore ankle stability. Surgical options include direct posterior buttress plating, which allows anatomic restoration of the articular surface and posteroinferior tibiofibular ligament, and indirect syndesmotic stabilisation using screws or suture-button constructs [4]. While the risks and benefits of each technique have been reported individually, there is limited comparative data on complication profiles between direct plating and syndesmotic fixation, including infection, malreduction, or fixation failure [5].

Postoperative rehabilitation, including weight-bearing, is also variable. Early mobilisation has been associated with improved functional recovery and fracture healing without increased fixation failure, yet delayed weightbearing remains common in UK practice, particularly in elderly or medically complex patients [6]. The interaction between fixation strategy and postoperative mobilisation following posterior malleolar fixation remains unclear [7].

The primary aim of this study was to compare posterior buttress plating and syndesmotic stabilisation for posterolateral posterior malleolar fractures with respect to postoperative complications. The secondary aim was to examine differences in postoperative weight-bearing practice, with the ultimate goal of guiding surgical decision-making for these injuries.

## Methods

### Study design and patient selection

A retrospective analysis was performed using a prospectively maintained trauma database at a Level 1 major trauma centre, identifying 1283 patients who underwent operative management of ankle fractures between January 2012 and June 2021. Radiographs of patients treated by fellowship-trained trauma or foot and ankle surgeons were reviewed to identify posterior malleolar fractures.

Exclusion criteria included distal tibial pilon fractures, ankle fractures without posterior malleolar involvement, skeletally immature patients, isolated syndesmotic injuries, follow-up less than eight weeks, and fixation methods other than posterior buttress plating or syndesmotic stabilisation (static screws or flexible suture-button constructs). After applying these criteria, 445 patients were included. The decision to proceed with operative fixation was made by the treating surgeon based on established indications for unstable ankle fractures, including loss of tibio-talar congruency, ankle instability, fracture displacement unsuitable for non-operative management, and overall fracture morphology.

### Radiographic assessment

Post-reduction lateral ankle radiographs were reviewed to determine fixation strategy and estimate posterior fragment size. The proportion of the articular surface involved was calculated by dividing the length of the posterior articular fragment by the total distal tibial articular surface on the lateral radiograph, as previously described. Although CT imaging is increasingly recognised as the gold standard for characterising posterior malleolar fracture morphology and guiding operative management, routine pre-operative CT was not universally performed throughout the study period. To permit standardised assessment across the entire cohort, radiographic measurements were therefore utilised for analysis. Where available, computed tomography (CT) was reviewed to classify posterolateral fragments according to the Mason classification system [8, 9].

### Data collection

Electronic patient records were used to collect demographic data, use of temporary external fixation, postoperative weight-bearing protocols, duration of follow-up, and subsequent complications. Patients were followed until discharge from outpatient review or death.

### Outcome measures

Postoperative complications included superficial or deep infection, loss of articular reduction, revision surgery, metalwork removal, and subsequent ankle arthrodesis. Infection was defined according to established fracture-related infection (FRI) guidelines, including clinical signs of infection, purulent drainage, wound breakdown, positive microbiological cultures, or confirmatory imaging or histopathology [10].

### Statistical analysis

Analysis was performed using JASP (Version 0.16; JASP Team, 2021). Continuous variables were compared with independent-samples t-tests and categorical variables with chi-squared tests. Statistical significance was defined as *p* < 0.05.

Multivariate logistic regression was used to identify factors independently associated with fixation strategy. For fixation choice, posterior buttress plating versus syndesmotic stabilisation was entered as the dependent variable, with age, sex, open fracture, temporary external fixation, posterior fragment size, and pre-operative CT as covariates.

### Ethical considerations

The study was assessed using the Health Research Authority decision tool and did not require formal ethical review. The project was registered with the local clinical audit department (AUDI002038).

## Results

A total of 1283 surgically treated ankle fractures were identified. Of these, 445 (34.6%) involved a posterior malleolar fracture. Following exclusion of patients with < 8 weeks of follow-up, 382 patients (86% follow-up rate) were included in the analysis. Thirty-eight patients (10.0%) underwent fixation with a tibiotalocalcaneal (TTC) nail, frequently utilising a retrograde femoral nail, reflecting the substantial number of elderly fragility and complex ankle fractures treated at our regional tertiary referral centre [11]. These patients were excluded from comparative analyses as TTC nailing represents a distinct treatment strategy from posterior buttress plating or trans-syndesmotic stabilisation.

Among included patients, 184 underwent posterior buttress plating and 111 received trans-syndesmotic stabilisation. Patients treated with a hindfoot nail (n = 38), no posterior malleolar fixation (n = 33), or alternative fixation methods (n = 16) were excluded. The mean follow-up duration was 10 months for the buttress plating group and 8.8 months for the trans-syndesmotic stabilisation group. Demographic characteristics are summarised in Table 1.

**Table 1:** Table of demographics of patients undergoing direct versus indirect fixation

Fixation method	N	Female, n (%)	Male, n (%)
Posterior buttress plate	184	109 (59%)	75 (41%)
Indirect (syndesmotic screw/tightrope)	111	54 (49%)	57 (51%)
Hindfoot nail	38	28 (74%)	10 (26%)
Not addressed	33	19 (58%)	14 (42%)
AP screw	3	2 (67%)	1 (33%)
PA screw	4	1 (25%)	3 (75%)
Fibular nail with syndesmosis screws	4	4 (100%)	0 (0%)
Frame	1	0 (0%)	1 (100%)
Other	4	2 (50%)	2 (50%)
Total	382	219 (57%)	163 (43%)

Postoperative complications are summarised in Table 2. Metalwork removal was more common following syndesmotic stabilisation (37.8% vs 10%, *p* < 0.001). This rate is consistent with published literature and largely reflects planned elective removal of syndesmotic screws, which remained common practice during the study period, rather than removal for complications such as infection or fixation failure. Unplanned removal due to infection or fixation failure was included in the revision or infection rates. There were no statistically significant differences between groups in rates of revision surgery, loss of reduction, or subsequent ankle arthrodesis.

**Table 2:** Postoperative complications in patients included in the comparative analysis (n = 295)

Variable	Direct fixation	Syndesmotic stabilisation	*P* value
Infection [n (%)]	28 (15.2%)	10 (9.0%)	0.123
Revision surgery [n (%)]	8 (4.3%)	2 (1.8%)	0.242
Ankle fusion [n (%)]	5 (2.7%)	1 (0.9%)	0.284
Loss of reduction [n (%)]	7 (3.8%)	3 (2.7%)	0.612
Removal of metalwork [n (%)]	19 (10.3%)	42 (37.8%)	< 0.001

Postoperative weight-bearing instructions were available for 270 patients in the comparative cohort. Overall, early weight-bearing (full weight-bearing, weight-bearing as tolerated, partial weight-bearing, or touch weight-bearing) occurred in 93 patients (34.4%). Early weight-bearing was more frequent in the buttress plating group (69/165, 41.8%) than in the trans-syndesmotic stabilisation group (24/105, 22.9%) (Table 3). Of 445 eligible posterior malleolar fractures, 382 had at least 8 weeks of follow-up and were included in descriptive analysis. Of these, 295 underwent either posterior buttress plating or syndesmotic stabilisation and formed the comparative study cohort.

**Table 3:** Comparison of weight-bearing status between the two groups

Variable	Posterior buttress plating (n = 184)	Syndesmotic stabilisation (n = 111)	*p*-value
Patients with WB data, n (%)	165 (89.7%)	105 (94.6%)	0.12
Early weight-bearing*, n (%)†	69 (41.8%)	24 (22.9%)	< 0.001

*Early weight-bearing was defined as full weight-bearing, weight-bearing as tolerated, partial weight bearing, or touch weight-bearing

†Percentages were calculated using patients with documented postoperative weight-bearing instructions in each treatment group (n = 165 and n = 105, respectively)

In a multivariable logistic regression adjusting for age, sex, open fracture, temporary external fixation, posterior fragment size (% articular surface and fragment length), and preoperative CT, buttress plating was associated with higher odds of early weight-bearing compared with trans-syndesmotic stabilisation (adjusted OR 2.81, 95% CI 1.12–11.46; N = 306).

## Discussion

This study found that postoperative complication profiles were broadly comparable between direct posterior malleolar fixation using buttress plating and indirect stabilisation with trans-syndesmotic fixation for posterolateral posterior malleolar fractures. No statistically significant differences were observed in revision surgery, loss of reduction, infection, or subsequent ankle arthrodesis. However, syndesmotic fixation was associated with substantially higher rates of metalwork removal, occurring in 37.8% of patients compared with 10% in the buttress plating group (*p* < 0.001), although most removals were elective and performed according to planned surgeons’ preferences rather than in response to complications.

These findings are consistent with previous reports demonstrating acceptable complication rates following direct posterior fixation. Forberger reported postoperative soft tissue complications in 5 of 45 patients (11%) treated with buttress plating through a posterolateral approach, with no revision surgery required and satisfactory reduction achieved in all cases [12]. Jeyaseelan et al. [13] similarly demonstrated improved functional outcomes after direct fixation, with a mean Olerud-Molander Ankle Score of 87 in the plating group compared with 74 after indirect syndesmotic fixation. However, implant removal was required in 23% of plated cases. In contrast, Mertens reported sural nerve paraesthesia in 18% of patients treated with posterior plating, suggesting that the risk of complications may also depend on surgical exposure, fracture morphology, and local soft-tissue factors [14].

Anatomical reduction of the posterior malleolus remains an important determinant of long-term ankle function. Residual articular incongruity greater than 1 mm has been associated with an increased risk of post-traumatic osteoarthritis. In contrast, fragments involving more than 25% of the distal tibial articular surface are linked to poorer long-term outcomes in several series [15, 16]. More recent evidence suggests that fracture morphology and syndesmotic attachment may be more clinically relevant than fragment size alone, particularly in posterolateral fragments that contribute substantially to syndesmotic stability [2, 17, 18]. Contemporary assessment of syndesmotic injury increasingly incorporates arthroscopic techniques, including needle arthroscopy, which allow direct visualisation of syndesmotic reduction and identification of associated chondral and osteochondral pathology [19]. These modalities may improve detection of subtle malreduction and facilitate intraoperative decision-making, although their routine use remains dependent upon local expertise and resource availability. Direct buttress plating offers the theoretical advantage of restoring both the articular surface and the attachment of the posteroinferior tibiofibular ligament [20]. Biomechanical studies suggest that posterior fixation restores approximately 70% of native syndesmotic stiffness, compared with around 40% using syndesmotic screws alone [21].

Despite these theoretical advantages, no significant reduction in major complications was observed in the present cohort. This suggests that indirect syndesmotic stabilisation remains a reasonable option in selected fracture patterns, particularly where direct posterior exposure is less favourable [17, 22].

The higher likelihood of early postoperative weight-bearing following buttress plating is clinically relevant. In this cohort, early mobilisation occurred in 41.8% of patients treated with buttress plating compared with 22.9% of those undergoing trans-syndesmotic stabilisation, with an adjusted odds ratio of 2.81 (95% CI 1.12–11.46). This association between buttress plating and an increased likelihood of early weight-bearing is a novel finding and likely reflects surgeon preference and confidence in the biomechanical stability of direct fixation rather than established evidence of superior clinical safety. As postoperative rehabilitation protocols were not standardised within this cohort, the observed relationship should be interpreted with caution. It may reflect perceived construct stability and surgeon preferences rather than a direct effect of the fixation strategy itself [20]. Biomechanical studies demonstrate that posterior buttress plating provides greater resistance to vertical displacement and reduced articular step-off under load compared with screw constructs, which may explain greater surgeon confidence in permitting earlier mobilisation following direct fixation [23–25].

However, contemporary clinical evidence increasingly suggests that early weight-bearing is safe irrespective of fixation method [26] [27]. Randomised controlled trials have demonstrated that early mobilisation at two weeks is non-inferior to delayed weight-bearing at six weeks following operative ankle fracture fixation, with comparable complication rates and improved early functional recovery [6]. The WAX trial, involving 561 patients across 23 UK hospitals, found no significant differences in loss of reduction, infection, or reoperation between early and delayed weight-bearing groups, and demonstrated a cost saving of approximately £60 per patient with earlier mobilisation. Similarly, the INWN trial showed that immediate weight-bearing in a walking boot resulted in superior early functional outcomes, with mean Olerud-Molander Ankle Score values of 43 versus 35 at six weeks (*p * = 0.005), together with cost savings of €798 per patient compared with six weeks of non-weight-bearing [28].

These findings suggest that differences in rehabilitation observed in the present study may be more reflective of surgeon behaviour than true construct-dependent restrictions [27]. Although posterior plating may offer greater biomechanical reassurance, current high-level evidence more broadly supports earlier mobilisation after stable ankle fixation [6, 29–31]. The wide confidence interval surrounding the adjusted odds ratio in this study reflects the relatively small number of early weight-bearing events and indicates some imprecision in the estimated effect; nevertheless, the direction of association remained consistent after adjustment for age, fracture severity, temporary external fixation, fragment size, and preoperative CT imaging.

The high rate of implant removal in the syndesmotic fixation group reflects variation in contemporary syndesmotic screw practice. Published series report removal rates ranging from 20 to 80%, with no clear functional advantage to routine removal and recognised risks including recurrent diastasis, infection, and need for further anaesthesia [32, 33]. Flexible fixation devices may reduce secondary procedures, although their use in this cohort was limited [22].

Several limitations should be acknowledged. The retrospective design introduces potential selection bias, particularly because the fixation strategy was determined by surgeon preference rather than by standardised criteria. Fracture morphology differed between groups, with larger fragments more commonly treated by buttress plating [34, 35]. Although multivariable regression adjusted for measured radiographic variables, unmeasured confounders such as smoking status, body mass index, diabetes, ankle dislocation, and surgeon experience may also have influenced treatment decisions and rehabilitation protocols. The observed higher infection rate in the buttress plating group (15.2% vs 9%, *p *= 0.123) did not reach statistical significance and may represent a type II error. In addition, postoperative weight-bearing protocols were not standardised and likely reflect variation in surgeon preference rather than a protocol-driven rehabilitation strategy [13]. The relatively short follow-up period limits assessment of longer-term outcomes, particularly post-traumatic osteoarthritis, which may not have been captured within the present study. Furthermore, CT imaging was not available in all cases, limiting fracture classification according to the Mason classification and potentially resulting in unrecognised morphological differences between treatment groups that may have influenced treatment selection and outcomes.

## Conclusion

Posterior buttress plating and trans-syndesmotic stabilisation demonstrated similar overall complication profiles in the treatment of posterolateral posterior malleolar fractures. No significant differences were observed in infection, loss of reduction, revision surgery or subsequent ankle arthrodesis.

These findings support the use of direct fixation as a safe treatment option for appropriately selected posterolateral posterior malleolar fractures. Whilst direct fixation was associated with earlier weight-bearing in this cohort, postoperative rehabilitation protocols were not standardised and likely reflect surgeon preference as well as perceived construct stability.

In the presence of a syndesmotic injury associated with a posterior malleolar fragment of sufficient size to permit fixation, direct fixation of the posterior fragment may restore syndesmotic stability and facilitate earlier mobilisation. However, treatment selection remains influenced by fracture morphology and surgeon preference. Further prospective comparative studies incorporating morphology-matched fracture groups, standardised rehabilitation protocols, longer-term follow-up, and patient-reported outcome measures are required to define the optimal fixation strategy more clearly.

## Data Availability

No datasets were generated or analysed during the current study.
