# Correction: Buttress plating versus trans-syndesmotic stabilisation for posterolateral malleolus fractures: outcomes with early weight-bearing

**DOI:** 10.1007/s00590-026-04965-x

**Published:** 2026-10-21

**Authors:** Katie Hutchinson, Georgina Crate, Christopher Fenner, Alex Trompeter, Nielsen

**Affiliations:** 1 St George’s University Hospitals NHS Foundation Trust, London, UK; 2 Kingston Hospital NHS Trust, London, UK; 3 St George’s, University of London, London, UK

**Correction to: European Journal of Orthopaedic Surgery & Traumatology (2026) 36:352** **https://doi.org/10.1007/s00590-026-04904-w**

In the original version of this article the author’s name Nielsen was incorrectly written as Dominic Nielsen.

The original article has been corrected.

